# The role of liver kinase B1 in tumor progression through regulation of lipid metabolism

**DOI:** 10.1007/s12094-022-02863-2

**Published:** 2022-07-27

**Authors:** Jialu Geng, Yanghe Zhang, Qingfei Meng, Hang Yan, Yishu Wang

**Affiliations:** Key Laboratory of Pathobiology, Ministry of Education, Jilin University, Changchun, 130021 China

**Keywords:** LKB1, Lipid metabolism, Tumor, AMPK

## Abstract

The somatic mutation of liver kinase B1 (LKB1) has been implicated in various tumors, which is reflected in the survival, proliferation, and metastasis of tumor cells. However, the regulation of LKB1 in lipid metabolism, a process that is involved in tumor progression is not completely clear. We conclude that LKB1 deficiency results in abnormal expression and activation of multiple molecules related to lipid metabolism which locate downstream of AMP-activated protein kinase (AMPK) or salt-induced kinase (SIK). Abnormal lipid metabolism induced by LKB1 deficiency contributes to the proliferation and metastasis of tumor cells through energy regulation.

## Introduction

Liver Kinase B1 (LKB1), also known as the serine-threonine kinase (STK) 11, is a tumor suppressor gene. LKB1 functions as a protein kinase which plays a vital role in maintaining energy homeostasis by phosphorylating and activating AMP-activated protein kinase (AMPK) family members [1]. LKB1 mutation is associated with the occurrence, development, and poor prognosis of several types of cancer, such as lung cancer, melanoma, cervical cancer, and hepatocellular carcinoma (HCC) [2–5].

Proliferation and metastasis of tumor cells involved in alteration of energy metabolism [6]. Although glucose and glutamine are important sources to maintain energy requirements for tumor cells [7], fatty acid oxidation (FAO) also provides potent metabolism support and possesses higher energy conversion efficiency [8]. In addition, excessive accumulation of lipid, which is stored in the form of lipid droplets, maintains the survival of tumor cells in energy-restricted condition [9]. LKB1 participates in the regulation of lipid synthesis, oxidation, and uptake in two ways. On the one hand, LKB1 activates downstream kinases, such as salt-induced kinase (SIK) and AMPK, which involve the activation and expression of several proteins related to lipid metabolism [10]. For instance, multiple key enzymes and transcription factors locate downstream of LKB1 and participate in de novo FA synthesis, such as sterol regulatory element-binding protein (SREBP), acetyl-CoA carboxylase (ACC), fatty acid synthase (FASN), and stearoyl-CoA desaturase-1 (SCD1) [11]. Upregulation of these FA synthesis enzymes was displayed promoting the development of several types of human cancer [12]. On the other hand, LKB1 regulates glucose metabolism via direct or indirect manners, such as the pentose phosphate pathway (PPP), which provides NADPH to lipid synthesis [13, 14]. Therefore, we focused on pathways under the regulation of LKB1 which involve lipid metabolism, and analyzed their role in the proliferation or metastasis of tumor cells. We are certain that the development and application of targeted inhibitors that process function to reverse abnormal lipid metabolism will contribute to improving prognosis in LKB1-deficient tumors.

## Lipid metabolism pathways under the regulation of LKB1

### LKB1-AMPK pathway

**Fig.1:**
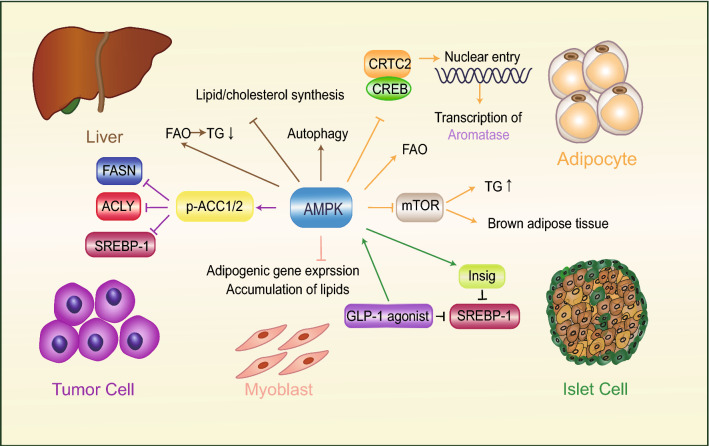
AMPK regulates lipid metabolism in multiple tissues and cells. Activation of AMPK reduces lipid accumulation in the liver, adipocyte, islet cell, tumor cell, and myoblast via modulating the expression of genes related to lipid metabolism, inhibiting activities of metabolic enzymes involved in lipid biosynthesis, and intensifying FAO and autophagy. *AMPK* AMP-activated protein kinase; *SREBP-1* sterol regulatory element-binding protein 1; *ACC* acetyl-CoA carboxylase; *FASN* fatty acid synthase; *TG* triglyceride; *FAO* fatty acid oxidation; *Insig* insulin-induced gene; *GLP-1* glucagon-like peptide-1; *mTOR* mechanistic target of rapamycin; *ACLY* ATP citrate lyase; *CRTC2* cAMP-regulated transcriptional coactivator 2; *CREB* cAMP response element binding protein

AMPK is an uppermost downstream kinase of LKB1, which functions as a central regulator of energy metabolism [15]. AMPK plays a vital role in energy metabolism and maintaining physiological functions in multiple types of cells and tissues (Fig. 1). For instance, AMPK mediates LKB1 deletion-induced enhancement of lipid-related gene expression in proliferative myoblasts [16]. Activation of AMPK significantly reduces hepatic triglyceride (TG) levels and stimulates the utilization of FA through restoring FAO [17] and inhibiting the de novo synthesis of lipid and cholesterol [18]. Activation of the LKB1-AMPK pathway also facilitates hepatocyte autophagy, which reduces lipotoxicity induced by lipid accumulation by blocking the formation of lipid droplets (LDs) [19]. Moreover, the AMPK-activated insulin-induced gene (Insig) is a protein that is bound with oxysterol in the endoplasmic reticulum (ER), which suppresses FA synthesis through inhibiting SREBP1c cleavage [20]. Liraglutide (LRG) is an agonist of Glucagon-like peptide-1 (GLP-1) receptor, which inhibits lipid synthesis by activating the AMPK-mechanistic target of rapamycin complex 1 (mTORC1)-SREBP1 pathway to ameliorate hepatic lipid accumulation [21].

AMPK negatively regulates ACC which mediates adipogenesis by catalyzing the formation of malonyl-CoA [22]. AMPK activation reduces the expression of FASN and ATP-citrate lyase (ACLY) and inhibits the activity of SREBP1, which suppresses tumor development in breast and prostate cancer [23, 24]. Together, AMPK regulates lipid metabolism by inhibiting lipid synthesis and promoting FAO, and AMPK deficiency-induced excessive lipid accumulation provides sufficient energy for the proliferation and metastasis of tumor cells. Notably, AMPK is also regulated by the ratio of AMP/ATP and calcium/calmodulin-dependent protein kinase levels [25, 26] Although LKB1 is responsible for regulating AMPK activation as the main upstream kinase, other signal pathways need to be considered when studying LKB1-AMPK pathway.

### LKB1-SIK pathway

SIKs are members of the AMPK superfamily, which functions through controlling phosphorylation and the subcellular localization of class IIa histone deacetylases (IIa HDACs) and cAMP-regulated transcriptional coactivators (CRTCs) [10]. Activation of the LKB1-SIK pathway suppresses tumor development, which had been confirmed in a mouse model of lung adenocarcinoma [27]. There were 38% upregulated genes which can be attributed to SIK1/3 inactivation in all upregulated genes induced by LKB1 deficiency in non-small cell lung cancer (NSCLC) [27]. Besides, LKB1-SIK3-HDAC4 signaling functions to repair the lipid solubility defect induced by LKB1 mutants in drosophila, which is a SIK3-dependent process that isn’t affected by activation of AMPK [28]. Further study suggested that LKB1-SIK3-HDAC4 had been regarded as a potential therapeutic target for acute myeloid leukemia [29].

LKB1-SIK2 regulates the phosphorylation of CRTC2, CRTC3, and HDAC4 in adipocytes [30]. Deletion of CRTC2 reduces the expression of SREBP1c, FASN, and ACC [30]. SIK2 not only determines the properties of white adipose tissue by controlling the activity of cAMP-response element-binding protein (CREB)-CRTC2-dependent transcription [31], but also promotes cholesterol synthesis by upregulating the expression of SREBP2 in ovarian cancer cells [32]. We consider that LKB1-SIK-CRTC/HDAC4 may act as a potential target for the inhibition of tumor growth.

### PPP affects lipid synthesis by providing NADPH

PPP produces ribulose-5-phosphate (Ru-5-P) and nicotinamide adenine dinucleotide phosphate (NADPH) [13]. Glucose-6-phosphate dehydrogenase (G6PD) is a rate-limiting enzyme of PPP, which is an important target of LKB1 [14]. Thus, LKB1 deficiency-induced upregulation of lipid synthesis can be attributed to intensifying PPP which provides material and facilitate the expression of genes related to FA synthesis.

PPP flux mediated by LKB1-AMPK-HDAC10-G6DP signaling had been shown to promote the growth of lung cancer cells [14]. However, LKB1-AMPK signaling is activated in breast cancer which reduces the level of G6PD transcription [33]. We speculated that upstream stimulators and tumor backgrounds underlie the different regulatory effects of LKB1 on G6PD. In the presence of glycolysis disorder, the levels of NADPH in LKB1-mutant A549 cells are higher compared to H1334 cells with normal LKB1 [34]. Nonetheless, Ru-5-P level was not changed in A549 cells with LKB1 overexpression or H1299 cell with LKB1 knocked down [14], which provides evidence that LKB1 regulates NADPH level through G6PD but Ru-5-P might be interfered with by other pathways. In addition, activation of the AMPK-SIRT1-p53 pathway increases the phosphorylation of ACC in A549 and NCI-H1299 cells [35], while downregulation of p53 increases G6PD activity in colon cancer [36], which leads us to establish a relationship between LKB1-AMPK signaling and p53-mediated G6PD regulation. Thus, further studies on the connection between LKB1, PPP, and tumor lipid metabolism are needed.

### CD36 promotes lipid uptake and storage

CD36 is an important FA transporter located on the surface of various types of cells. LKB1 deficiency results in increased expression of CD36 and decreased expression of ATP binding cassette (ABC) transporter, which leads to dysfunction of cholesterol uptake, de novo synthesis, and outflow [37]. In lapatinib-resistant breast cancer cells, a gene set enrichment analysis (GSEA) and Gene Ontology (GO) enrichment analysis revealed a significant enrichment of lipid-related pathways [38]. However, upregulated of CD36 was principal, and there were no significant differences in FASN and carnitine palmitoyltransferase-1A (CPT1A) levels [38], which provides a hypothesis that CD36 mediated lipid uptake and storage, but not lipid synthesis of FAO, plays an irreplaceable role in metastatic of breast cancer cells. Deletion or inhibition of CD36 limits the uptake of FAs from the tumor microenvironment and reduces adipocyte-mediated invasion and migration of prostate cancer cells [39]. In addition, CD36 is closely related to the survival and metastasis of numerous tumors, such as lung cancer, bladder cancer, breast cancer, and melanoma [40]. Therefore, improvement of disorder lipid metabolism by blocking CD36 is an effective therapeutic strategy for inhibiting tumor development, which deserves to be expected in clinical transformation.

### Other downstream lipid regulators of LKB1

**Fig. 2:**
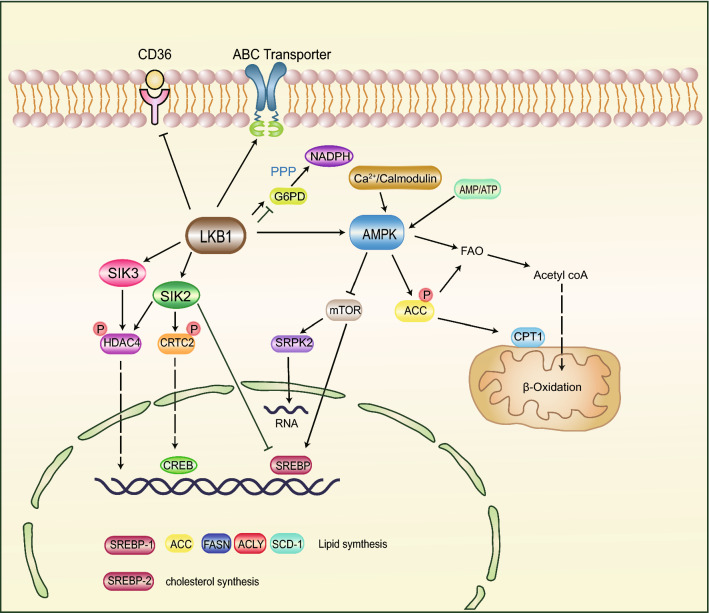
Pathway mediated regulation of LKB1 in lipid metabolism. LKB1 inhibits SREBP-induced lipid synthesis and facilitates fatty oxidation through activating AMPK. LKB1 also phosphorylates HDAC and CRTC via activating SIKs, which further inhibit the expression of lipogenic genes. LKB1 reduces lipid uptake by regulating lipid membrane transporters. LKB1 facilitates and suppresses lipid synthesis via upregulating and downregulating PPP in tumor cells with different background genotypes. LKB1 liver kinase B1, *AMPK* AMP-activated protein kinase, *SIK* salt-induced kinase, *mTOR* mechanistic target of rapamycin, *ACC* acetyl-CoA carboxylase, *FAO* fatty acid oxidation, *SREBP* sterol regulatory element-binding protein, *FASN* fatty acid synthase, *SCD1* stearoyl-CoA desaturase 1, *ACLY* ATP citrate lyase, *PPP* pentose phosphate pathway, *G6PD* glucose-6-phosphate dehydrogenase, *NADPH* nicotinamide adenine dinucleotide phosphate, *SRPK2* serine/arginine-rich protein kinase 2, *HDAC4* histone deacetylase 4, *CRTC2* cAMP-regulated transcriptional coactivator 2, *CREB* cAMP response element-binding protein, *ABC* ATP binding cassette, *CPT1* carnitine palmitoyltransferase 1

Although regulation of lipid metabolism by LKB1 is a complex process (Fig. 2), energy storage is the ultimate purpose which is provided to sustain survival under energy stress conditions. Thus, LKB1 deficiency-induced abnormal lipid metabolism is displayed as enhancement in FA uptake and FA synthesis, and suppression in FAO. The LKB1-mTOR signaling inhibits differentiation of adipocyte and TG accumulation in brown adipose tissue by activating FAO and downregulating peroxisome proliferator-activated receptor γ (PPARγ) [41, 42]. Serine/arginine-rich protein kinase 2 (SRPK2) is required for de novo lipogenesis, which is under the regulation of mTOR [43]. Acetyl-CoA mediates a negative regulatory loop in the LKB1-AMPK-ACC pathway, which is a substrate of FA synthesis but participates in LKB1 acetylation [44]. We summarized that LKB1 mediated regulation of lipid metabolism processes complexity, further experimental investigations need to be conducted in different types of cancers because of the varied genetic background.

## Upstream regulated molecules of the LKB1 pathway

### SIRT

Sirtuin 1 (SIRT1) is required for AMPK activity. Bouchardatine is an effective inhibitor of adipogenesis, which reprograms lipid metabolism by activating SIRT1-AMPKα-ACC in rectal cancer [45], further study revealed that LKB1 mediates this signal transduction process [46, 47]. Notably, SIRT1 promotes adipocyte lipolysis in mesenteric adipose tissue but has the opposite inhibitory effect in epididymal adipose tissue [48], which illustrates certain restricted conditions, such as tissue types or genetic background, affect the role of SIRT1 in the regulation of lipid metabolism. Even so, we considered that the targeted SIRT1-LKB1 pathway remains a potential therapeutic strategy for tumors with explicit genetic background.

### cAMP/PKA

cAMP-dependent protein kinase A (PKA) functions transcriptional regulation of genes related to adipogenesis. The melanocortin system plays a key role in controlling appetite and adipogenesis [49]. An investigation showed a pathway that α-Melanocyte-stimulating hormone (α-MSH)-cAMP-PKA-extracellular signal-regulated kinase (ERK) -LKB1-AMPK in hypothalamus cells, which provides a therapeutic target for lipid metabolic diseases [50]. In addition, cAMP also activates the LKB1-AMPK-SREBP pathway, which suggested that increased cAMP regulates lipid metabolism by reducing SREBPs expression [51].

PKA-LKB1 signaling pathway inhibits 3-hydroxy-3-methylglutaryl-CoA reductase (HMGCR), ACC, and SREBP-1 through activating AMPK in insulin-resistant HepG2 cells [52]. Although LKB1 has been considered a tumor suppressor traditionally, it functions as the facilitation of tumorigenesis in thyroid cancer as the downstream mediator of PKA [53]. On the one hand, PKA opposes the negative regulation of lipolysis through phosphorylating AMPKα1 at ser173 which inhibits the activation of AMPK mediated by LKB1. On the other hand, activation of PKA directly stimulates adipocyte lipolysis, which triggers a negative feedback mechanism of AMPK. The appropriate utilization of the negative feedback loop balance between PKA and AMPK contributes to the precise regulation of lipolysis response [54]. The complex regulatory mechanism between PKA, LKB1, and AMPK provides support for maintaining metabolic balance, and may also promote tumor development, which needs to further study.

### Hormones and inflammation

Hormones and inflammation are important factors that affect the development of cancers. Given that aromatase mediates hormone synthesis and several inflammatory mediators are classed as lipid, abnormal lipid metabolism also participates in the development of tumors through regulating hormones and inflammation. Prostaglandin E-2 (PGE2), an inflammatory mediator, inhibits the activity of the LKB1-AMPK pathway and regulates aromatase in breast adipose stromal cells [55]. In addition, it has been indicated that leptin induces aromatase expression by inhibiting the LKB1-AMPK pathway and subsequently nuclear translocation of CRTC2, while adiponectin has the opposite effect to leptin [56]. Vitamin D has been found to inhibit aromatase expression and local estrogen synthesis in tumor cells and adipose tissues through increasing LKB1 activity in response to the adverse effect of obesity that contributes to breast cancer growth [57]. In conclusion, the targeted LKB1 pathway may provide effective intervention for the high-risk obese people who need to prevent the occurrence and development of breast cancer more than others.

## LKB1 deficiency-induced abnormal metabolism facilitates tumor progress

### ACC-driven lipogenesis supports cell survival and metastasis of tumors

ACC is a major downstream lipid regulator of LKB1-AMPK signaling. ACC accelerates FAO in two CPT1-dependent manners. On the one hand, phosphorylation of ACC inactivates the ability to mediate malonyl-coA production, which promotes FAO with increased CPT1A content [58]. On the other hand, the formation of a complex between ACCα and CPT1A attenuates in the case of glucose deficiency, which boosts FAO by facilitating the transport of FA [59]. Upregulation of ACCα and p-ACC are predictors of recurrence and poor survival of tumors [59, 60]. Thus, we considered that ACC has the potential to act as a detection index, a tumor prognostic marker, or a therapeutic target.

### SREBP1 maintains lipid synthesis and tumor cell viability

SREBP locates downstream of LKB1, and AMPK or SIK mediates the regulation of LKB1 for SREBPs. Activation of SREBPs regulates the expression of genes related to the key enzymes in cholesterol synthesis and FA synthesis [12]. Multiple types of tumors display activation of the mTORC1-SREBP pathway, which facilitates lipid synthesis [61]. In addition, SREBP1 is essential for the viability of cancer cells under hypoxic conditions [62]. SREBP1 is associated with the degree of malignancy and prognosis of tumors, which has been identified in metastatic melanoma [63]. Inhibition of SREBP1 increases the sensitivity of tumor therapy [64, 65], which inspires us to further develop selective SREBP1 inhibitors to suppress the development of tumors.

### SCD promotes tumor growth and proliferation

SCD supports lipogenesis and desaturation of tumor cells under the reduced supply of exogenous lipids, which is important for cell viability and proliferation of tumors [66]. In addition, SCD expression is related to the survival time of patients, which can be used as a marker of tumor prognosis [67, 68]. The expression level of SCD1 in lung adenocarcinoma was higher than in adjacent normal tissues [67]. High expression of SCD usually indicates the presence of advanced disease in breast cancer and prostate cancer [66]. SCD1 also promotes the migration and invasion of tumor cells [67]. Thus, targeted inhibition of SCD has the potential to improve prognosis in tumors with high SCD expression.

### Promoting effect of CPT1A on cancer

CPT1 is a rate-limiting enzyme of FAO, whose isozyme CPT1A deficiency inhibits the invasion and growth of radiotherapy-resistant breast cancer by reducing FAO [69]. A gene transcriptional splicing variant CPT1Av2 expressed in breast cancer cells interacts with HDAC1 molecules, which regulates the epigenetic inheritance of genes involved in tumor-associated cell death and invasion pathways [70]. In addition, the anti-tumor mechanism of mTORC involves the reduction of CPT1A expression and lipid catabolism [71]. Down-regulated CPT1A expression reduces lipid utilization by attenuating lipid catabolism, which contributes to the downregulation of cancer-related gene expression and apoptosis of the tumor cells [69–71]. These results revealed that targeting FAO contributes to the regulation of tumor energy metabolism, and CPT1A is a potential metabolic target in cancer therapy.

### ATGL affects tumor energy storage

Adipose triglyceride lipase (ATGL) is a rate-limiting enzyme in the triglyceride hydrolysis cascade, whose expression is reduced in malignant tumors [72]. The absence of ATGL induces the development of lung tumors in animal models and low levels of ATGL are associated with poor survival in patients with ovarian cancer, NSCLC, breast cancer, and stomach cancer [72]. In addition, the lipolysis activity of ATGL is crucial for the distant metastasis of tumor cells, which depends on the gradual release of stored free FA by LDs [73]. ATGL is required for the adipocyte-mediated proliferation of breast cancer cells, which uptake energy from adipocytes through the transfer of fatty acids [74]. Taken together, ATGL is considered to exert a tumor-inhibiting effect due to its deficiency in malignant tumors. However, as mechanisms of lipid metastasis provide great energy support for tumor cells, ATGL involves in tumor aggressiveness, which demonstrates further studies are required to confirm the effects of ATGL on tumor growth.

## Application: targeted inhibitors of LKB1 pathways on liver diseases and tumors

### Inhibitors activate the LKB1 lipid regulatory pathways

The tankyrase antagonist G007-LK was shown to play a dual role in lowering blood glucose and inhibiting the development of lung cancer by activating the LKB1-AMPK pathway [75]. A pan-Pim kinase antagonist AZD1208 with anti-cancer activity enhances LKB1 phosphorylation and reduces p-ACC, which resists adipogenesis [76]. ONC201 is an antagonist of dopamine receptor D2, which exerts anti-tumor effects in both obese and non-obese LKB1^fl/fl^/p53^fl/fl^ mice with endometrial tumors. Mechanically, results from metabonomic analyses showed that ONC201 upregulated lipid synthesis [77]. Honokiol is a potential leptin antagonist, which induces SIRT1/3-mediated activation of LKB1-miR-34a signaling to antagonize tumorigenesis induced by leptin in breast cancer [78].

## Activators target LKB1 downstream to release liver diseases

Several types of liver diseases relate to abnormal lipid metabolism [79]. We summarized the active factors of LKB1 and possible therapeutic effects in lipid metabolism disorders in liver diseases (Table 1). Notably, natural plant extracts and their derived compounds are beneficial to correct metabolic disorders, which applicated may exert energy stress on tumor cells to achieve suppressed effect.

**Table 1:** The factors acting on LKB1-mediated pathways in lipid metabolic diseases [80–85]

Diseases	Factors	Mediated signalling pathways	Effects
NAFLD	Paeoniflorin α/γ-mangostin	LKB1-AMPK-SREBP1c/ FAS	Inhibiting lipid synthesis and enhancing FAO
		SIRT1-LKB1-AMPK-ACC/CPT1A	
ALD	Gentiopicroside	P2X7 receptor/IL1β-LKB1-AMPK-SREBP/ACC/PPARα	Reducing lipogenesis and promoting lipid oxidation
NASH	DMY	SIRT1-LKB1-AMPK-ACC	Inhibiting lipid synthesis and enhancing FAO
Liver fibrosis	HIF-1α	lncRNA-H19-LKB1-AMPK	Accelerating FAO and LDs degradation
Excessive TG	LTA	LKB1-AMPK-ACC1/SREBP1c-FAS	Enhancing oxidation and transport of lipids and reducing the synthesis of TG, cholesterol, and lipid accumulation
		LKB1-AMPK-HMGCR	

*NAFLD* Non-alcoholic fatty liver disease, *ALD* alcoholic liver disease, *NASH* non-alcoholic steatohepatitis, *TG* triglyceride, *FAO* fatty acid oxidation, *LDs* fat droplets, *PUE* Pueraria lobata, *SIL* Silybum marianum, *DMY* dihydromyricetin, *HIF-1α* hypoxia-inducible factor-1α, *LTA L* theanine

### Inhibitors target LKB1 downstream to treat tumors

Several targeted inhibitors of the LKB1 pathway which relate to lipid regulation have been conducted in extensive preclinical trials in tumors. ND646, an ACC allosteric inhibitor, exerted a strong inhibitory effect on NSCLC [86]. Selective SREBP1 inhibitors reduce the expression of FASN, HMGCR, and SCD1, which decrease lipid synthesis in pancreatic cancer cells [87]. Sorafenib targets SCD1 through the ATP-AMPK-mTOR-SREBP1 signaling pathway and induces the death of HCC cells [88]. CPT1 inhibitor ST1326 counteracts the proliferation of chronic lymphocytic leukemia cells by blocking FAO and depleting acetyl-CoA in the cytoplasm [89].

Drug resistance is a practical problem in tumor treatment. *ACC* mutants with AMPK phosphorylation site deficiency were reported to protect head and neck squamous cell carcinoma cells to escape cetuximab-induced growth inhibition [90]. The incidence of *BRAF* gene mutations in melanoma is as high as 40–50%, but the therapeutic effect is not optimal when targeted BRAF. Owing to SREBP1-dependent continuous lipogenesis being a key mechanism of drug resistance induced by the BRAF mutant, simultaneous inhibition of SREBP1 enhances the sensitivity of melanoma [91], which suggests that identifying the mechanism of resistance and then synergistic use of agents will better sensitize and combat tumors.

## Conclusion

LKB1 demonstrates a satisfactory ability to inhibit tumor progression, whose mutation is a mark of poor prognosis of tumor. Different cell sources and genetic backgrounds affect the expression and function of LKB1. However, LKB1 deficiency-induced abnormal lipid metabolism which is mediated by the disordering of transcriptional regulatory factors was proved to be detrimental. This review focuses on the role of LKB1-mediated pathways, which affect tumor progression through disturbing lipid metabolism. Because of the drug resistance of existing inhibitors, bimolecular targeted inhibitors and chemotherapy or radiotherapy combined with targeted inhibitors improve the therapeutic effect of tumors. In-depth studies of LKB1 are contributed to combining multiple targets for detection, which improves the accuracy of tumor hierarchical classification and stratification based on LKB1 expression. We considered that the selection of appropriate target inhibitors for sensitive tumor types may improve malignant lipid metabolism, which has the potential to inhibit tumor proliferation and metastasis.

## Abbreviations

LKB1: Liver kinase B1
STK: Serine-threonine kinase
HCC: Hepatocellular carcinoma
FA: Fatty acid
AMPK: AMP-activated protein kinase
SIK: Salt-induced kinase
PPP: Pentose phosphate pathway
SREBP: Sterol regulatory element-binding protein
ACC: Acetyl-CoA carboxylase
FASN: Fatty acid synthase
SCD1: Stearoyl-CoA desaturase 1
TG: Triglyceride
FAO: Fatty acid oxidation
LDs: Lipid droplets
Insig: Insulin-induced gene
ER: Endoplasmic reticulum
GLP-1: Glucagon-like peptide-1
LRG: Liraglutide
mTORC1: Mechanistic target of rapamycin complex 1
KO: Knockout
ACLY: ATP citrate lyase
HDACs: Histone deacetylases
CRTC: CAMP-regulated transcriptional coactivator
CREB: CAMP response element binding protein
NADPH: Nicotinamide adenine dinucleotide phosphate
Ru-5-P: Ribulose-5-phosphate
G6PD: Glucose-6-phosphate dehydrogenase
ABC: ATP binding cassette
GSEA: Gene set enrichment analysis
GO: Gene Ontology
CPT1A: Carnitine palmitoyltransferase 1A
PPAR: Peroxisome proliferator-activated receptor
SRPK2: Serine/arginine-rich protein kinase 2
ATGL: Adipose triglyceride lipase
NSCLC: Non-small cell lung cancer
SIRT1: Sirtuin 1
NAFLD: Non-alcoholic fatty liver disease
PKA: CAMP-dependent protein kinase A
ERK: Extracellular signal-regulated kinase
HMGCR: 3-Hydroxy-3-methylglutaryl-CoA reductase
HSF1: Heat shock factor 1
PGE2: Prostaglandin E2
